# Neurocognitive development in HIV-positive children is correlated with plasma viral loads in early childhood

**DOI:** 10.1097/MD.0000000000006867

**Published:** 2017-06-08

**Authors:** Valentin Weber, Daniel Radeloff, Bianca Reimers, Emilia Salzmann-Manrique, Peter Bader, Dirk Schwabe, Christoph Königs

**Affiliations:** a Department of Pediatrics and Adolescent Medicine; b Department of Child and Adolescent Psychiatry, Psychosomatics and Psychotherapy, University Hospital, Goethe University, Frankfurt am Main; c Department of Child and Adolescent Psychiatry, Psychotherapy and Psychosomatics, University Hospital Leipzig, Leipzig, Germany.

**Keywords:** cART, neurocognitive development, perinatally HIV-infected children, viral load

## Abstract

Because of neurocognitive impairments in perinatally human immunodeficiency virus (HIV)-infected children and adolescents, this study aimed to demonstrate the effect of plasma viral loads and early initiation of sufficient combined antiretroviral therapy (cART) on neurocognitive development.

In total, 14 perinatally infected HIV-positive children (median age 8.24 years [range: 6.0–16.74]) receiving lopinavir/ritonavir (LPV/r)-based ART underwent neurocognitive testing using the Wechsler Intelligence Score for Children, 4th Edition (WISC-IV). All 14 patients participated in a pharmacokinetic study in which they were hospitalized for an entire day. As a child's ability to concentrate varies over the course of the day, all tests were performed in the morning.

The patients’ neurocognitive development did not significantly differ from the normative collective pattern for any of the following composite scores that were examined: full-scale intelligence quotient (IQ) (mean: 106.5, *P* = .1060), verbal comprehension index (mean: 106.0, *P* = .1356), perceptual reasoning index (mean: 106.0, *P* = .1357), working memory index (mean: 106.3, *P* = .1171), and processing speed index (mean: 98.1, *P* = .6313). The overall full-scale IQ scores were significantly higher in children who began ART within the first year of life (*P* = .0379), whereas low lopinavir/r plasma levels (*P* = .0070) and high viral load area under the curves (AUCs) in the first 3 years of life, but not later, significantly correlated with reduced neurocognitive performance (Spearman *r*^2^ = −0.64, *P* = .0278).

In this cohort of cART treated HIV-positive children and adolescents, neurocognitive performance correlated with early and sufficient viral load suppression within the first 3 years of life.

## Abstract 2

Supplemental Digital Content is available in the text

## 1 Introduction

An untreated human immunodeficiency virus (HIV) infection in children leads to cognitive and motor deficits, even without the occurrence of HIV encephalopathy (HIVE), and is associated with increased mortality and permanent neurocognitive deficits.^[1–7]^ The different criteria for and definitions of neurological deficits in adults and children make it difficult to uniformly determine the prevalence and incidence of HIV-associated neurological deficits. Currently, a shift to milder deficits is also associated with the availability of combined antiretroviral therapy (cART) and a reduced progression of deficits.^[8]^ Although the improvement in HIVE symptoms by cART is undisputed, the influence and extent of cART on neurocognitive abilities and development remains unclear.^[9]^ HIV-associated central nervous system (CNS) diseases are particularly common in countries with limited treatment resources. Particularly, in these parts of the world, perinatally infected children and adolescents show significantly impaired neurocognitive performance compared with the respective uninfected population.^[10]^ Despite advances in pediatric HIV-therapy, HIV-infected children undergoing therapy often have difficulties in school.^[11,12]^ High cluster of differentiation 4 (CD4) cell counts do not prevent the development of neurocognitive deficits.^[13]^ Early and severe clinical manifestations increase the risk of permanent neurocognitive deficits. More severe HIV-related neurological manifestations in early life may impact the organization and myelination of white matter microstructure.^[14]^ Because deficits cannot be fully compensated, infancy remains a window of opportunity to make a beneficial impact on the child's neurocognitive development.^[7,15,16]^ Although cART effectively reduces the viral load in the cerebrospinal fluid and clinical benefits are obvious,^[17–19]^ the effect of cART on neurocognitive abilities remains to be clarified. All studies published thus far commonly demonstrate impaired neurocognitive development of different degrees that are influenced by the timing of cART regimens. The current study aimed to demonstrate the effect of the early initiation of sufficient cART on neurocognitive development in a group of young children and adolescents perinatally infected with HIV.

## 2 Materials and methods

### 2.1 Study population

In this single-center, non-randomized, non-controlled clinical trial, the eligibility criteria were as follows: (1) perinatal HIV-infection; (2) age 6 to ≤16 years; (3) fluency in German; (4) available virological, immunological, and cART histories; and (5) lopinavir/ritonavir (LPV/r) based ART-regimen with available pharmacokinetic (PK) data. All children and adolescents participated in a PK study with different formulations of LPV/r. Tablet-PK data were collected prospectively, and liquid/capsule-PK data were collected retrospectively.

The appropriate Ethics Committee of the Goethe University of Frankfurt, Faculty of Medicine, Germany and the relevant competent authorities approved the work and proposed study design (EudraCT: 2010–021622–35); the study was conducted in accordance with the principles of the Declaration of Helsinki. Legal guardians provided written consent and study participants provided written assent before participation in the study.

### 2.2 Outcome measures

Neurocognitive testing was uniformly performed using the German edition of the Wechsler Intelligence Score for Children, 4th Edition (WISC-IV), which is a measure of neurocognitive abilities in childhood and adolescence for individuals aged 6 to 16.11 years. The WISC-IV uses 10 core subtests to determine the values of the following 4 domains of cognitive functioning: verbal comprehension (VC), perceptual reasoning (PR), working memory (WM), and processing speed (PS). The raw scores are converted into age-related subtest standard scores for each domain and then combined to yield a full-scale intelligence quotient (FSIQ). The reference mean for the FSIQ and each subtest is 100, with a standard deviation (SD) of 15.^[20]^

All examinations were performed in the morning to minimize the influence of diurnal variations in performance. Only children and adolescents who had not been subject to WISC-IV testing in the preceding year were included in the study to exclude the influence of practice effects on the test results. The inquiries showed that none of the children had ever undergone WISC-IV testing.

### 2.3 Statistical methods

The results of the WISC-IV were tested for normal distribution using D’Agostino & Pearson omnibus normality test and then compared with the reference mean and SD using the unpaired *t* test. The clinical parameter data collected retrospectively and the time of the initiation of therapy were compared with FSIQ and the prospectively collected pharmacokinetic parameters using the Mann–Whitney *U* test. A 2-sided *P* < .05 was considered statistically significant. As a measure of the exposure of the body to the virus, the viral AUC (viral copies∗years/mL) was determined for the first 3 years of life of each child. The correlation between viral exposure and FSIQ was determined using the Spearman rank correlation.

Despite the nature of the study with a predefined patient population, a samples size calculation was performed to value the significance of this study. The sample size n = 13 has a beta power = 0.90 with 2-sided *α* = 0.05 to detect a minimal relevant delta difference equal to 15 between the mean of the sample and the referent value FISQ = 100 with a SD of 15.

All pharmacokinetic analyses were performed with WinNonLin (Pharsight Corporation, Cary, NC). GraphPadPrism 4 (GraphPad Software, Inc., La Jolla, CA) was used for statistical analyses.

## 3 Results

Neurocognitive testing was performed on a total of 14 of the 15 children and adolescents enrolled in the study. The remaining adolescent suffered from severe disabilities following an untreated HIV infection and encephalopathy in early childhood. Based on these disabilities including the limitations in speaking the patient was excluded from the neurocognitive testing.

The remaining 14 perinatally HIV-infected children (5 women, 9 men) had a median age of 8.24 years (range: 6.0–16.74) at the time of neurocognitive testing. The median CD4 cell count at the time of the neurocognitive testing was 1751 cells/μL (median, range: 211–3820) and 35.15% (median, range: 14.2–53.6). Eleven children had suppressed viral loads (<50 copies/mL) at the time of testing, and the remaining 3 children had viral loads of 74, 2150, and 5070 copies/mL. Therapy was initiated in this cohort at the median age of 1.34 years (range: 0.11–8.78). cART was initiated according to German guidelines.^[21]^ The leading reasons cART was initiated in the early treated group were age (<12 months, n = 2), recurrent pneumonia, CMV coinfection, HIVE, and immunodeficiency (n = 2), and, in the later treated group, the reasons were severe immunodeficiency (n = 5), wasting, and HIVE. The median viral load at the initiation of therapy was 5.18 log_10_ copies/mL (range: 2.0–6.41), the median CD4 count was 1360/μL (range: 38–3230), and CD4% was 25.50% (range: 3.5–64.8). The detailed baseline characteristics are shown in Table 1. The study was linked to a PK study that analyzed LPV/r plasma levels; all participants received LPV/r twice daily at the licensed dose in combination with 2 nucleoside reverse transcriptase inhibitors, including lamivudine/emtricitabine, abacavir, zidovudine, or tenofovir.

**Table 1:**
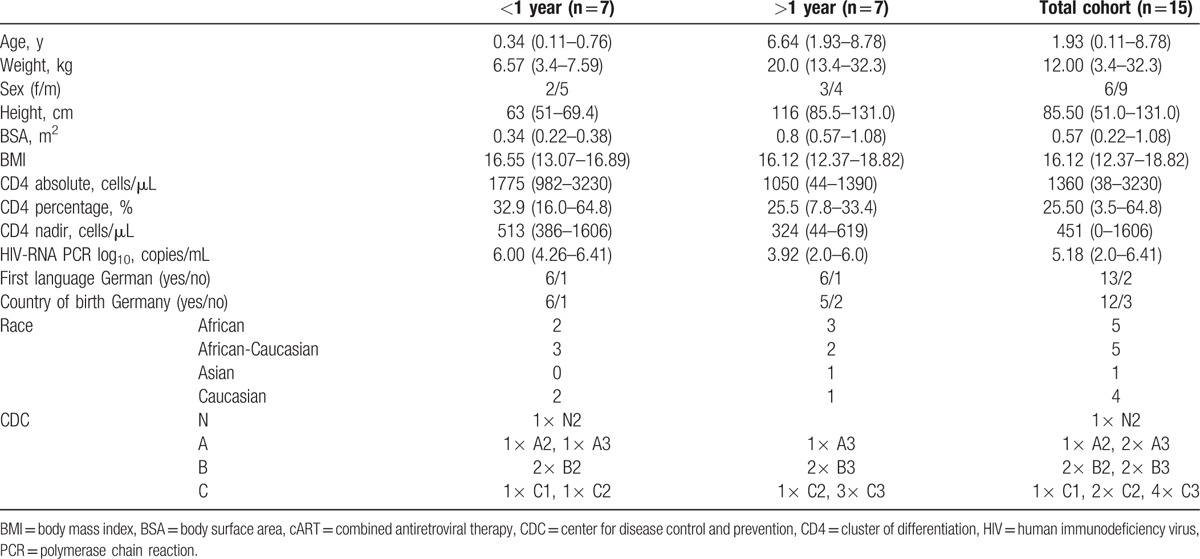
Patient characteristics at the start of cART according to the treatment group (median [range]).

Testing for a normal distribution using D’Agostino & Pearson omnibus normality test, no significant differences to a normal distribution of the results for FSIQ (*P* = .6832), verbal comprehension (*P* = .3745), perceptual reasoning (*P* = .4382), working memory (*P* = .8194), and processing speed (*P* = .7990) were assumed.

**Figure 1:**
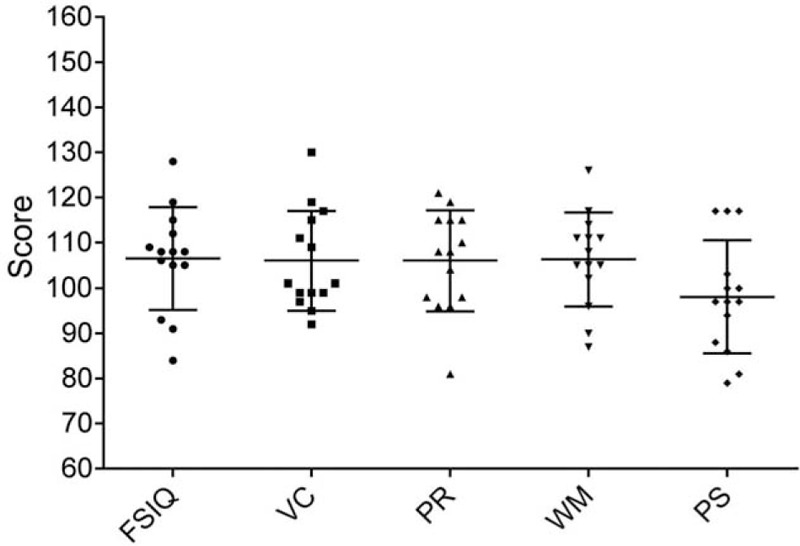
Results of neurocognitive testing. FSIQ = full-scale intelligence quotient, PR = perceptual reasoning, PS = processing speed, VC = verbal comprehension, WM = working memory. The mean and SD are shown. SD = standard deviation.

The average overall intelligence quotient (IQ) was within the normal range (106.5 ± 11.33) and did not differ significantly from the published WISC-IV-normative sample (*P* = .1060).^[20]^ The scores for the domains of verbal comprehension (106.0 ± 11.0, *P* = .1356), perceptual reasoning (106.0 ± 11.2, *P* = .1357), working memory (106.3 ± 10.4, *P* = .1171), and processing speed (98.1 ± 12.5, *P* = .6313) also did not differ from the WISC-IV-normative sample. Figure 1 shows the results of the neurocognitive testing for the different batteries. The individual results are shown in Supplementary Table S1.

FSIQs were correlated with the individual cART history. Initiating antiretroviral therapy in the first year of life significantly correlated with higher overall IQ values (patients 1, 2, 3, 6, 11, 12, 13 vs. 4, 5, 7, 8, 9, 10, 14; median FSIQ 112 vs. 105, *P* = .0379) compared with children starting at any older age.

**Figure 2:**
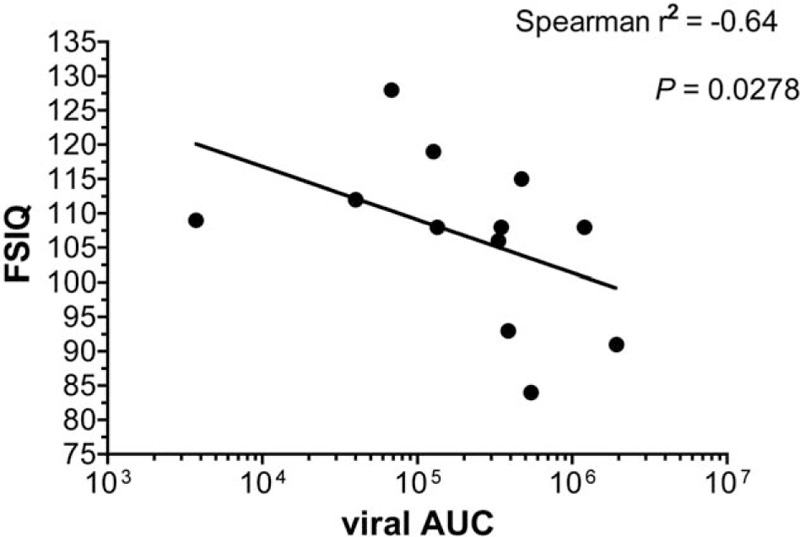
Correlation between FSIQ and viral AUC for the first 3 years of life. FSIQ = full scale intelligence quotient, viral AUC = viral area under the concentration–time curve (viral copies∗first 3 years/mL).

The viral load data over the patients’ lifetime were available at least every 3 months since diagnosis. The results (copies/mL) were plotted against age to calculate the viral area under the curve (AUC) (see supplementary Fig. S1) and to evaluate the impact of viral exposure over their entire life from diagnosis to the study on the neurocognitive development. Higher viral loads over time expressed as AUC values in the first 3 years of life significantly correlated with lower overall IQ values (Spearman *r*^2^ = 0.64, *P* = .0278) (Fig. 2). This correlation lost significance if the AUC and FSIQ were correlated over the total lifespan of the patient or after the age of 3. Likewise, there was a significant difference in FSIQ when the group was analyzed comparing children starting cART <3 years with viral load suppression and children starting cART >3 years or not achieving viral load suppression (patients 1, 2, 9, 11, 12, 13, 14 vs. 3, 4, 5, 6, 7, 8, 10, *P* = .0041).

The PK data from the 2 different time points were also correlated to the FSIQ values. For the first PK (PK1), at the median age of 1.76 years (range: 0.28–8.9), minimum concentrations of lopinavir below the median of 2775 ng/mL (20–4900 ng/mL) correlated with lower FSIQ scores (*P* = .0070), whereas the maximum lopinavir concentration of 9595 ng/mL (0.9398) and the lopinavir AUC of 81,200 ng∗12 h/mL (*P* = .1218) did not correlate with lower or higher neurocognitive performances within the group. There was no correlation between the FSIQ and PK data (PK2) that were obtained within the current study. Clinical and pharmacokinetic data at the time of PK1 and PK2 are summarized in Table 2.

**Table 2:**
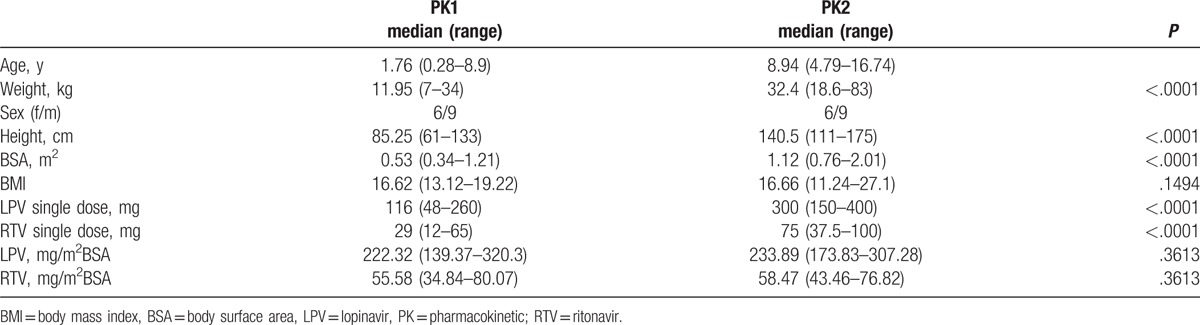
Patient characteristics at the first and second PK.

## 4 Discussion

In this uniform and closely observed but small cohort, the neurocognitive examination of the patient collective by means of the WISC-IV showed an age-appropriate development of neurocognitive performance in perinatally HIV-infected children and adolescents receiving effective cART. The calculated FSIQ scores of the entire patient group were within the normal range. The results indicate an unimpaired cognitive development in this uniform and effectively treated group of perinatally infected children.

No neurocognitive test results from an earlier time point prior to the study were available for the enrolled patients, thus excluding any training effect and simultaneously preventing the formation of any conclusions on the dynamics of neurocognitive performance over time. It has been reported in the literature that HIV-associated neurocognitive deficits are not fully compensated in patients after initiating ART.^[22]^ In an early terminated trial no additional benefit of cART was seen for a CNS-targeting therapy.^[23]^ Most studies report on neurocognitive effects after a relatively short timeframe of treatment: the participants in the current study were children and adolescents of at least 6 years of age thus reflecting a longer period of cART influencing neurocognitive development.

A major difference between horizontally HIV-infected adults and vertically infected children and adolescents is that HIV also affects the brain after it has fully developed in horizontal infection, whereas the virus affects the brain during a very crucial time of CNS development in perinatally infected children.^[24,25]^ In addition, HIV-exposed, but uninfected, children may develop deficits.^[26]^

Importantly, 1 of the 15 children and adolescents enrolled in the pharmacokinetic study was not tested in this study due to a severe HIV encephalopathy in early childhood related to an untreated HIV infection with high viral loads in the first years of life. The neurocognitive impairment in this child is consistent with the published data on the effects of untreated infection on neurocognitive abilities mentioned above and further underscores the importance of early and sufficient antiretroviral therapy.

The incidence of HIV-encephalopathy was reduced dramatically after the introduction of highly active antiretroviral therapy for perinatally infected children and adolescents.^[27]^ Further neurological conditions were also reduced as shown for epilepsy in infected children who started cART early in life.^[28]^ A recently published study showed that 23.3% of 287 Jamaican HIV-infected children involved in the study developed HIV-encephalopathy at a median age of 1.6 years. Again, the importance of early recognition of neurocognitive deficits and the treatment of the infection was emphasized.^[29]^ In a further study, 284 HIV-positive children and adolescents from Thailand and Cambodia were divided into the following 2 groups: “early treatment” and “late treatment.” “Early” was defined as therapy beginning simultaneously at baseline irrespective of age, whereas in the second group, study therapy was started only if the CD4 cell count percentage fell below 15% or an AIDS-defining event had occurred. Regardless of the study arm, after 144 weeks of follow-up, the entire HIV-positive patient group (median age 9 years) showed significantly decreased neurocognitive performance compared with uninfected children. Although the group of patients who began ART early had a significantly higher CD4 cell counts after 144 weeks compared with patients who began their therapy at the later time point (33% vs. 24%, respectively; *P* < .001) and the proportion of children with viral loads below the limit of detection also differed (91% vs. 41%, respectively), no improved neurodevelopmental outcome was noted for the group starting at baseline in this study.^[16]^ All children were older than 1 year at the start of therapy, which is consistent with the observation of this study that the start of treatment before the age of 1 year results in a good neurocognitive outcome. Similar results were reported in a recently published Dutch cohort as follows: the neurocognitive function was within the normal ranges for most children (29/35) but reduced compared with age-matched controls, and 6/35 children showed a cognitive impairment. This impairment was also correlated with structural deficits in the brain. In this cohort, not all children were on current cART, and, again, most children started therapy at >1 year.^[30,31]^ No improvement in neurocognitive function was observed in children with “earlier” therapy in a study in the United States; however, the mean age at the start of treatment was 5.85 years.^[32]^

In the present study, “early” treatment initiation was defined at a much younger age (below the age of 1 year) and compared with any later start of therapy. An early therapy according to this definition correlated significantly with higher FSIQ values likely due to a lower burden of viral replication, emphasizing the crucial role of cART in the first year of life.

Furthermore, viral AUCs from the time of diagnosis (or availability of viral load measurements) until the neurocognitive testing were established to more precisely analyze the viral burden over life compared with analyzing individual time points, such as the peaks of viral loads. High viral AUCs in the first 3 years of life significantly correlated with poorer neurocognitive performance. However, when comparing the viral load AUCs after the age of 3 or over the children's entire lifetime, this significance was lost. Consequently, a correlation between FSIQ and the results of the early PK was only observed after the start of cART, but this correlation was not observed with the PK performed at the time of the neurocognitive testing later in life. A significant correlation was only seen for LPV/r trough concentrations (*C*_min_) but not for LPV/r maximum concentrations (*C*_max_) and LPV/r AUC did not correlate with neurocognitive performance.

This finding might explain the poorer neurocognitive outcome in other patient cohorts as therapy was mainly initiated at an older age. Thus, the influence of viral loads and effective cART on neurocognitive function was less pronounced.

These findings illustrate the importance of a successful suppression of viral replication over time for later neurocognitive outcomes, particularly in the first year of life. This period of early childhood appears to be a crucial window for a therapeutic intervention to prevent neurocognitive deficits. This finding is consistent with the physiological development of a child's brain. During the first years of life, dramatic development and an increase in brain mass are observed.^[25]^

A further study showed better age-appropriate neurocognitive development performance when starting treatment within the first 3 months compared with a delayed start of therapy in perinatally infected infants.^[15]^ This finding is consistent with the finding of the current study. However, in contrast to this study, the scores were not in the ranges of uninfected children potentially due to the different methods and parameters being tested. In addition, testing was performed in much younger children at the median age of 11 months, which does not account for the further development and influence of cART and, therefore, renders the groups not comparable.^[15]^

The correlation of LPV/r *C*_min_ early in life and neurocognitive function is consistent with the observation of the correlation only between early viral AUC and neurocognitive outcomes. The correlation between plasma levels of antivirals and neurocognitive performance indicates a possible role of the central penetration effectiveness score (CPE) in the outcome. Antiretroviral substances with a high CPE score showed advantages in the prevention of neurocognitive disorders and mortality in adults.^[33]^ Positive correlations between cognitive abilities and the suppression of viral RNA in the cerebrospinal fluid have been published, but only a few effects have been reported to be significant.^[34]^ In contrast, in a recently published pediatric study, CPE-sores showed no association with neurocognitive outcomes in school-aged children.^[35]^ Similar findings were noted in an adult population shortly after the initiation of ART in a randomized trial.^[23]^ A recently published study involving adult patients showed diminished neurocognitive performance in patients whose antiretroviral medication had a CPE >2.5 compared with those patients whose antiretroviral medication had lower CPE values.^[36,37]^ In the current study, all therapy regimens had a CPE >3 as lopinavir/r itself already has a CPE-score of 3.^[36]^ Nevertheless, no effect of CPE-scores was observed in children and adolescents.^[35]^

The results of this work are somewhat limited due to the small sample size. The small number allowed a very extensive follow-up over the children's lifetime and the establishment of parameters allowing the quantification of steady viral exposure.

Perinatally, HIV-infected children of this cohort who are sufficiently treated with cART in their first year of life do not exhibit impaired neurocognitive development. This early treatment correlates with higher neurocognitive performance in youth and adolescence and, therefore, further supports effective cART very early in life.

## Acknowledgments

The authors wish to thank the patients and their families for participating in the study.

## Supplementary Material

Supplemental Digital Content
