# Supplementary material for: Neurocognitive development in HIV-positive children is correlated with plasma viral loads in early childhood
Source: Medicine (Baltimore). 2017 Jun 8;96(23):e6867. doi: 10.1097/MD.0000000000006867 (PMC5466203; doi:10.1097/MD.0000000000006867)
Supplement: Supplemental Digital Content [file medi-96-e6867-s001.docx]

**Supplementary Figure S1: Viral loads of individual patients over life-span**

The individual viral load measurements (copies/ml) for the individual patients (different symbols) are plotted against the patients’ age. The solid line indicates the median.

**Supplementary Table S1: Results of Neurocognitive Testing**

(FSIQ: full scale intelligence quotient, VC: verbal comprehension, PR: perceptual reasoning, WM: working memory, PS: processing speed)

| Patient | FSIQ | VC | PR | WM | PS |
| --- | --- | --- | --- | --- | --- |
| 001 | 119 | 130 | 115 | 111 | 97 |
| 002 | 108 | 99 | 108 | 102 | 117 |
| 003 | 93 | 92 | 104 | 96 | 86 |
| 004 | 105 | 101 | 108 | 105 | 100 |
| 005 | 91 | 95 | 98 | 87 | 81 |
| 006 | 108 | 115 | 98 | 111 | 100 |
| 007 | 105 | 99 | 96 | 108 | 117 |
| 008 | 108 | 119 | 96 | 114 | 94 |
| 009 | 106 | 97 | 115 | 105 | 103 |
| 010 | 84 | 99 | 81 | 90 | 79 |
| 011 | 115 | 111 | 119 | 126 | 88 |
| 012 | 128 | 109 | 121 | 117 | 117 |
| 013 | 112 | 117 | 115 | 105 | 97 |
| 014 | 109 | 101 | 110 | 111 | 97 |
